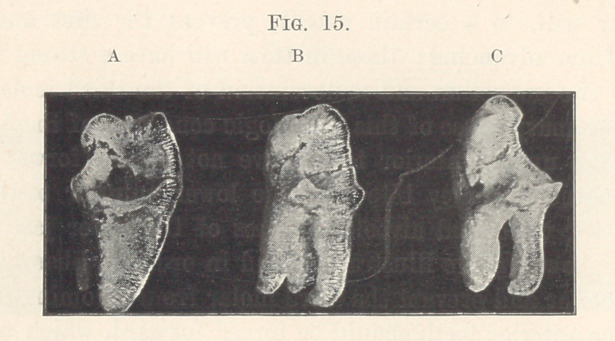# Retarded Eruption of the Teeth: Their Liberation or Extraction

**Published:** 1905-01

**Authors:** M. H. Cryer

**Affiliations:** Philadelphia


### International Dental Journal.

Vol. XXVI. January, 1905. No. 1.

### Original Communications.[1]

### RETARDED ERUPTION OF THE TEETH: THEIR LIBERATION OR EXTRACTION.[2]

BY M. H. CRYER, M.D., D.D.S., PHILADELPHIA.

In order to fully understand this subject one should first be thoroughly familiar with the typical positions of the teeth when entirely erupted and in normal occlusion with those of the opposing jaw. It is also necessary to have a complete knowledge of the internal anatomy of the alveolar process. Therefore, a few illustrations showing the typically erupted teeth, with their occlusion, and the internal anatomy of the alveolar processes will be shown.

Fig. 1 is a side view made from an almost perfect skull of a white woman. The teeth are fully erupted and are almost typical in their position and occlusion. It is evident there has been but little interference with the nutrition of either the jaws or the teeth of this subject. It will be noticed that the mental foramen is on a line drawn vertically downward from between the premolar teeth. This is quite typical and will be again referred to. It is

[1] The editor and publishers are not responsible for the views of authors of papers published in this department, nor for any claim to novelty, or otherwise, that may be made by them. No papers will be received for this department that have appeared in any other journal published in the country.

[2] Read at the fifty-fifth annual session of the American Medical Association, in the Section on Stomatology, at Atlantic City, June 7 to 10, 1904.

the external opening of a small tube communicating with the inferior dental canal. For this small tube, which I have described in previous papers, the name "mental tube" is suggested. Its internal opening into the inferior dental canal is situated in the

Fig. 1.

Side view of a typical skull with typical occlusion of the teeth.

movable cancellated tissue near the apex of the root of the canine. If the skull of an infant be examined at birth it will be found that the mental tube, or short canal, passes directly outward. nearly opposite the lower portion of the germ of the canine tooth. Then, again, if the skulls of children of various ages, up to adult life, be examined, it will be found that the inlet of this mental tube has been carried forward along with the cancellated tissue of the teeth, while the outlet has, apparently, moved backward, the distance varying according to the age of the child until adult life is reached.

The position of the first molar during its early development was immediately below the upper or inner angle of the jaw. As it increases in size and the other molars are developed, the entire mass of cancellated tissue containing the teeth move forward, the upper portion a little farther than the lower, as is indicated by the curvature of the trabecular and small cribriform tubes passing from the main tube or canal to the roots of the various teeth. To accommodate this growth the mandible proper—the cortical portion—enlarges interstitially as this process is carried on. Interference with this forward movement of the teeth in the cancellated tissue would have a tendency to arrest the enlargement of the jaw, no matter whether the interference be caused by artificial means or by pathologic conditions.

The development of the alveolar process of the upper jaw is somewhat different. While the teeth are developing and erupting the process extends outward and forward without the extension of the maxillæ which is observed in the mandible. When the teeth are finally lost in extreme old age and the alveolar process of both

FIG. 2.

A B

Under view of two skulls. A, from a subject about twenty years old; B, from one well advanced in years.

jaws is resorbed, the extended rim of the mandible remains, while the upper jaw recedes until the roof of the mouth becomes very small, as is well illustrated in Figs. 2 and 3.

Fig. 4 is made from the skull of a child about six years old.

The external plates of the alveolar process of the upper and lower jaws have been removed exposing the roots of the deciduous teeth

Fig. 3.

Side view of the two skulls shown in Fig. 2.

Fig. 4.

Skull of a child about six years of age, showing all the deciduous teeth with their roots and crowns of nearly all the permanent teeth.

and the crowns of the developing permanent teeth, except those of the lower third molar and the upper first, second, and third molars.

In the majority of children's mouths, at about the age of six, barring accident or decay, the upper and lower teeth (deciduous) are typical in their arrangement in the alveolar process; they are also typical in their occlusion. While pathologic and other disturbances seem to interfere but little in the arrangement of the deciduous teeth, they greatly influence the placing of the permanent teeth.

The positions of the non-erupted teeth shown in Fig. 4 vary in regard to their depth in the bone. Some are deeply set, for example, in this instance the upper canine, while others are quite superficially placed, as illustrated by the lower canine. Others still are found located between the roots of the deciduous teeth, as the crowns of the premolars in the figure. If we take the positions of the developing permanent teeth into consideration, it is comparatively easy to understand why they should often be retarded in their eruption or deflected out of their typical course. If the deciduous teeth are interfered with in such a manner that their physiologic functions are impaired, they, in turn, will interfere with the physiologic functions of the tissue surrounding the permanent teeth.

Local or general pathologic conditions often cause the alveolar arch to become narrowed or shortened, which, in turn, will have more or less influence on the eruption of the permanent teeth. Malocclusion of the teeth is often accompanied by impaction, as will be shown in some of the illustrations. Occasionally supernumerary teeth, odontoma or odontoceles may cause impaction of the teeth.

### ORDER OF IMPACTION.

My experience has been that the frequency of impacted teeth is as follows: First, the lower third molar; second, the upper canine; third, the upper third molar; fourth, the upper central incisor; fifth, the lower second premolar; sixth, the upper second premolar; seventh, the lower canine. The first and second groups of this classification will, without doubt, be accepted by all familiar with the subject under discussion. In the museum of the Dental Department of the University of Pennsylvania the following specimens will be found: Twelve impacted lower third molars, nine impacted upper canines, two of which are in one jaw; two impacted upper third molars, both in the same jaw; two impacted lower second premolars.

Examination of Fig. 4 makes apparent reasons for this order of impaction. It will be seen that the germ of the lower second molar is well back and partly within the ramus of the jaw. The germ of the lower third molar is still farther upward and backward. As these teeth are developed and the jaw grows, the teeth and the cancellated tissue pass forward between the U-shaped cortical bone. If this sliding forward and downward of the tooth be interfered with by reason of inflammatory phenomena within the substance of the jaw, causing the cancellated and cortical portions to become adherent, the already erupted teeth will be prevented from yielding slightly to the eruptive force of the moving molar, and there will be no room for this tooth to slide into its proper position. The lower portion of the capsule is more liable to become retarded or fixed than the upper; consequently, in such a case the upper portion or crown of the tooth is carried forward and downward, causing it in many cases to take a horizontal position. In some instances it is turned directly upside down (see Fig. 5).

If the position of the germ of the upper canine be examined, it will be found on a higher level and deeper in the bone than the other teeth. The first premolar is erupted about three years before the canine and often closes in towards the lateral, erupted five years previously, especially if the deciduous canine has been lost early. Under ordinary circumstances the canine will be forced into a fairly typical position, but if any inflammatory condition of the jaw has been manifested the bone may become firm and the canine more or less impacted. Similar conditions can be predicated of nearly all impacted teeth.

Impacted permanent teeth should, as a rule, be either liberated or extracted. When the impacted tooth can be brought into a useful position through extraction of supernumerary teeth, or by the removal of other causes impeding its eruption, the necessary steps for its liberation should be taken. If left in their impacted state these teeth are liable to prevent the proper nourishment of other teeth and also to interfere with the healthy nutrition of the surrounding tissue. They may press on the branches of the trifacial nerve, producing neuralgia, not only locally, but in remote parts, and through reflex action they may cause various disturbances in and about the head and face. They are liable to bring about inflammatory conditions of this region, produce cellulitis in the tissues of the mouth, neck, throat, and temporo-mandibular articulation, interfere with deglutition, etc. They may cause other teeth to become impacted, or even cause malocclusion of other teeth. Then, again, parts of the roots may penetrate into the maxillary sinus or the nasal chambers, under which conditions, if they become devitalized, they are liable to cause the infection of these cavities.

These four illustrations (Figs. 5, 6, 7, 8) are made from dried specimens. They will give some idea of the most common form of impacted upper canines and lower third molars.

Fig. 5 is from a dried specimen belonging to Dr. T. M. Whitney, of Honolulu. It shows an inverted impacted lower third molar,

Fig. 5.

An inverted impacted lower third molar.

the crown of which is partly erupted in the submaxillary fossa. This tooth would be rather difficult to extract in the living subject. From the appearance of the illustration I would judge that it would have to be extracted through the submaxillary triangle.

Fig. 6 shows the anterior surface of an impacted canine tooth resting near the anterior surface of the bone. This tooth could have been diagnosed by the use of an excavator, as the crown was quite superficially covered.

Whenever canine teeth are missing from the arch there is good ground to suspect that they are impacted somewhere within the jaw, unless there is satisfactory evidence that they have been extracted. This is not to be assumed in the case of a missing upper lateral incisor or third molar of either jaw, and occasionally the premolars, all or any of which are often missing through non-development. As an example, I recall the case of a patient about thirty-five years of age, from whose arch the two upper second premolars are missing, and who claims that they have not been ex-

Fig. 6.

An impacted canine tooth.

tracted. As he suffered from neuralgia in the anterior portion of the maxillæ, I thought that these teeth might be impacted somewhere within the jaw, but a careful exploration with instruments and radiographs, taken at different angles and by several experts, failed to show any evidence of the missing teeth. The failure of these methods of exploration leads me to believe that the teeth in question have never developed.

The extraction of a tooth, such as Fig. 6, would not be difficult. A longitudinal incision could be made over the crown of the tooth, and then by passing a very small spiral osteotome around the crown one would be enabled to pass a universal elevator between tooth and bone. The tooth could then be forced outward; if room could be made so that a small pair of forceps could be used it might be better to use them instead of the elevator.

Occasionally I have found that the bone surrounding such teeth has become very dense and closely adherent to the tooth. Under these conditions the tooth is liable to break, if great care is not exercised, and sometimes it is necessary to use a small spiral osteotome to remove the surrounding bone nearly to the apex of the tooth before it can be extracted.

Fig. 7 shows two impacted canines in the roof of the mouth. They were covered principally by bone which extended down nearly

Fig. 7.

Two impacted canine teeth.

to the point of the crown. These teeth had caused the devitalization of the left first and second premolars and the right first premolar, also of the lateral incisor of the left side. The apex of one of the teeth penetrated the maxillary sinus.

It is more difficult to extract canine teeth when in this position than when situated as shown in Fig. 6, as the danger of causing an opening between the mouth and nose, or mouth and antrum, is greater. It is a good plan to remove the bone from the lingual surface of the tooth, so the tooth can be carried slightly downward and inward without danger of fracture. Usually there is not room to extract it in the line of its axis before it strikes the other teeth or alveolar process.

Fig. 8 shows a common form of impacted lower third molar.

The anterior cusps of the tooth are often interlocked against the concave distal portion of the second molar. In order to extract such teeth it is often necessary to cut these cusps away with either a carborundum disk or an osteotome. When this has been done the instrument can be passed under the tooth, between it and the bone, or both, until the elevator can be passed into the opening. By this

Fig. 8.

A common form of impacted lower third molar.

means the tooth can usually be removed in a comparatively short time.

The following are from practical cases:

Case I.—A patient was suffering from neuralgia on the right side of the face. The molar teeth apparently had been extracted from that side of the mandible, but on a closer examination a slight elevation was found about in the position of the right second molar, and by passing an excavator into the enlargement a tooth could be felt. An X-ray picture confirmed this diagnosis. The tooth was removed and the neuralgia ceased. In the X-ray picture the condyloid process is seen plainly resting against the under surface of the eminentia articularis.

In all the radiographs I have seen of this region, when the mouth was wide open, the condyloid process has been found in this position, indicating that the external pterygoid muscles acting together when the angle is comparatively fixed by other muscles, among which are the masseter and internal pterygoid, serve to assist in separating the jaws, by drawing the upper part of the ramus forward, which compels the anterior portion of the mandible to drop, opening the mouth.

CASE II.—Is a patient of Dr. Dray, of Philadelphia. This patient was suffering from neuralgia of the right side of the face. An X-ray picture showed an impacted lower third molar with the crown resting well forward against the distal root of the second molar. In such cases the resorption of this root often takes place, producing neuralgia and devitalization of the tooth. There was a great probability that the second molar was diseased and causing the neuralgia. It was, therefore, thought advisable to extract it, which proved the diagnosis to be correct.

CASE III.—Fig. 9 is made from a plaster cast taken from the mouth of a young woman about twenty-five years old in the practice of Dr. S. Merrill

FIG. 9.

Made from a plaster cast taken from the mouth of a young woman about twenty-five years old in the practice of Dr. S. Merrill Weeks, of Philadelphia, showing an impacted lower third molar.

Weeks, of Philadelphia. The central incisors are erupted with their cutting edges pointing slightly inward instead of outward, probably due to some pathologic condition of the deciduous incisors. The alveolar process around the incisors is harder than normal, which condition prevented these teeth from being carried outward during the eruption of the others. They were thus locked against the incisors of the lower jaw, causing these and other lower teeth to be held back to a greater or less degree, which in turn would cause the impaction of the lower third molars. The right central is directed more inward than the left and in proportion the right lower third molar was more deeply impacted than the left lower third molar. The left impacted tooth was extracted about a year before the casts were made. The right one was interlocked against the second molar. The occluding surface of this tooth was cut away by the use of a carborundum disk, which allowed the crown to rise slightly, as is shown in the illustration, thus making the tooth more prominent and easier to extract.

Fig. 10 is a radiograph taken from a cleaned specimen of the right side of the lower jaw, showing the teeth in position in the cancellated tissue.

FIG. 10.

An impacted second premolar and a third left molar.

One might well imagine that a modern orthodontist had moved the first molar " half its width backward" or held it in such a manner that it could not advance. Whether done by mechanical appliance or by pathologic changes, the tooth was held and impaction resulted. If the cancellated tissue be examined, as seen in the X-ray picture, it will be noticed that it is more dense around the first and second molars than anteriorly to these teeth. As the result of an inflammatory condition the cancellated tissue has become united with the cortical bone, thus making another factor in preventing its sliding forward. It will be noticed that the roots of the molar teeth are also thickened by the overaction of the cementoblasts caused by this inflammatory condition.

The inferior dental canal or cribriform tube is slightly deflected from its true course below the roots of the impacted third molar, and is also slightly deflected downward below the roots of the second molar.

It will be noticed that the second premolar stands below the occluding line of the other teeth. It has evidently been retarded in its eruption, perhaps through premature loss or devitalization of the second deciduous pre-

Fig. 11.

molar. In such cases these roots are resorbed very slowly and often cause inflammatory conditions. It is possible that this was one of the primary causes of the non-eruption of the third molar.

Fig. 12.

Case IV.—Fig. 11 is from a photograph of a plaster cast of the jaw of a young man about twenty-two years old. Two impacted upper third molars were diagnosed by the use of an excavator. The teeth were afterwards extracted by first removing the overlying tissue and then using small forceps.

Fig. 12 is from a photograph taken of the same cast (Fig. 11), with the extracted impacted teeth placed near the tuberosity of the jaw.

Case V.—A patient had some neuralgic trouble within the ear, and after having excluded several possible causes, the teeth were suspected, as the upper first and second molars appeared to be sensitive, and a radiograph was taken which indicated an impacted upper left third molar. At this time the patient was referred to me by Dr. George Darby. When one becomes accustomed to examining X-ray pictures it is not difficult to detect a shadow of the crown of a tooth in the region where the upper third molar might be impacted, but this picture gave but a slight indication as to the depth of its occluding surface. No idea was possible as to whether it was near the buccal surface of the alveolar ridge or on the lingual surface. The roots of the tooth, their number, shape, and position, were not shown in the radiograph. All of this practical surgical diagnosis had to be learned by other means. In this case a careful exploration was made with an excavator, and the position of the crown of the tooth was practically located. After the tissue covering the crown of the tooth had been cut away the tooth was grasped with small forceps. The firmness of the tooth indicated that the roots were crooked and held by bone harder than normal. By carrying the handle of the forceps in the line of least resistance, which was outward, backward, and upward, the roots were unlocked from under the over-calcified bone.

Fig. 13 is made from four photographs of the tooth after extraction. A shows the occluding or grinding surface with the points of the roots ex-

Fig. 13.

A B C D

tending outward. B shows the upper surface, or the root end, with the four roots spread outward, approaching a horizontal line. C shows the distal surface, with the hook-like form of the buccal roots, and D shows the anterior surface. It may be interesting to know that the ear has improved since the extraction, and that the other molars appear to have lost their sensitiveness, indicating that the tooth was interfering with the nerve supplying these teeth and through reflex action with the ear.

Fig. 14 was made from a plaster cast of a patient of Dr. G. Marshall Smith, of Baltimore, who has kindly permitted me to use it in this paper. The patient is a girl thirteen years old. Fig. 14 is a side view of a plaster cast of the teeth and alveolar processes. The teeth are undersized, the incisors and canines are too nearly vertical or do not flare enough, especially in the lower jaw. The upper teeth are all erupted, for this age, except the second molars, which are ready to erupt. In the lower jaw they are all

Fig. 14.

The second deciduous premolar is not in position.

through except the second premolar, which is impacted. The space for this tooth is very narrow, in fact, not sufficient to allow it to take its proper place in the jaw. The description of the teeth on one side of the jaw may also be applied to those of the other side.

If the cancellated tissue in the region of the premolar has become solidified and attached to the cortical bone, this impacted premolar will, to a certain extent, prevent the first and second molars from advancing; these in turn will have a strong tendency to cause the impaction and malocclusion of the third molar.

The primary cause of this pathologic condition of the lower jaw is that the upper anterior teeth have not moved forward in the usual manner, as they bite over the lower anterior teeth, consequently the teeth and alveolar process of the lower jaw are held back, as shown in the illustration, and in order to liberate the second premolar and prevent the third molar from becoming impacted, the upper anterior teeth should be forced forward, which will allow correction to be made in the lower jaw.

Case VI.—A young man about twenty-one years old, who has suffered from neuralgia of the left side of the mandible. On examination with an excavator, impacted lower right and left one being somewhat broken. The history of the case is that part of the crown of the left third molar has been broken away in an endeavor to extract the tooth, leaving the pulp exposed. A radiograph showed that the crown was deformed, also that the anterior buccal cusp was apparently interlocked under the second molar. By careful examination with an excavator it was found that both of the anterior cusps were so far down in the tissue that a disk could not be used to remove them. The patient being etherized, a mouth-gag was placed in position and a portion of the soft tissue was removed with a small knife. The revolving spiral osteotome was placed within the broken crown, or into the pulp-chamber, cutting almost through the remainder of the crown, and between it and the bone a space was made, partially in the bone, which allowed the point of a universal elevator to pass between the tooth and the jaw.

I seldom use the forceps to remove a tooth after loosening it with the elevator. In using the elevator on the left side, as in this case, it is operated with the right hand, the surgeon standing on the left side of the patient. The left forefinger is placed in the mouth by the lingual side of the tooth, and the thumb is placed on the buccal side of the second molar. This gives steadiness to the jaw and reduces the risk of slipping. As the tooth is raised from its socket the forefinger is placed so as to bring the tooth out of the mouth. If the tooth to be removed is on the right side, the elevator should be used with the left hand, if possible (the surgeon standing on the right side). If the operator must use the elevator with his right hand, he should, however, manage to guard and steady the parts with his left hand.

Fig. 15.

A B C

Fig. 15 is made from three photographs of the tooth after extraction. A shows the outer or buccal side of its roots, in about the same position as when in the jaw. The distal cusps were broken away in a former endeavor to extract it. The greater portion of the crown was cut away with the surgical engine. On the side of the tooth there is a groove extending backward, downward and inward, cut by the osteotome. It was along this groove that the elevator was forced under the tooth, causing the slight portion of the crown that remained to fracture. In B the tooth is turned slightly outward, in order to show three roots and the line of fracture which liberated the tooth. In C the tooth is turned on its buccal surface, showing the two anterior cusps which were locked under the distal surface of the second molar.

DISCUSSION.

*Dr. Edward A. Bogue,* New York City.—What does Dr. Cryer mean by "premolar"?

*Dr. Cryer.*—A tooth that is in front of the molar tooth.

*Dr. Bogue.*—Dr. Cryer in his paper used the term "premolar" for a deciduous tooth. I am sorry to hear such misuse of terms. He also stated that Dr. Angle says that if the sixth-year molars are correct in their occlusion all the rest will be right. If he will make further reference to Angle's book he will find that he states that the largest part of irregularities are to be found when the principal molar is in correct, or nearly correct, occlusion. I want to acknowledge my indebtedness to Dr. Cryer; I never hear him without benefit; at the same time, if any defect in his teaching occurs I want to call attention to it, that it may be corrected. As I understand his remarks in regard to impacted third molars he favors extraction. I have a case on hand now in which by tying a small grass line between the impacted third molar and the second molar below I am bringing that third molar to a proper occlusion. I have done this a number of times.

*Dr. Cryer.*—I said "liberated or extracted."

*Dr. Bogue.*—I am glad to see that Dr. Cryer's remarks tend in two directions. He advocated the forward development of both jaws. I agree with Dr. Cryer, whose experience is so much greater than mine, that one should be wary of the pressure backward.

*Dr. M. L. Rhein,* New York City.—Dr. Cryer's idea about the correction of the irregularity early in life and the sacrifice of the third molar later in life is one with which I thoroughly agree. It is expected in these cases of degeneration which cause a lack of space in the mandible itself that there will not be sufficient room for the sixteen teeth to come properly into position. His conclusions, so far as they can be put into practice, I think are the proper ones; that under these circumstances it will most likely be necessary to sacrifice this third molar, and it is the one that can best be sacrificed. I sympathize with all the work that has been done for the preservation of thirty-two teeth in the arch, and especially against the loss of any of the teeth forward of the third molar. There is such a thing as going to extremes on both sides of this question, and if there are any four of these thirty-two teeth to be lost the ones that can be spared with least detriment to the patient are the third molars without the occlusion suffering. In the case of the young Baltimore girl, there seemed to be little question as to what to do. It comes in the category named of gaining sufficient space between the first molar and the first bicuspid. If separation is made there Nature will cause that bicuspid to erupt without any difficulty. The undue prominence of the chin and the lack of bone development above it shows to me that it will not make the chin any more prominent. There is plenty of room above the chin for the osseous development, if this forward movement is brought about. I have had a number of cases in which after space was provided Nature brought the teeth into position.

*Dr. Mihran K. Kassabian,* Philadelphia.—The dental and medical professions have derived equal benefits from the introduction and application of the X-rays. The diagnostic value of the rays in cases of unerupted teeth is shown by Dr. Cryer's cases. The ordinary methods of probing with an explorer did not locate the absent teeth, which in some cases were bicuspids and third molars. These had insinuated themselves deeply between other teeth, and the alveolar process was so dense that nothing but the X-ray could locate them. I employ two methods for skiagraphing dental conditions, the intraoral method and the extraoral or buccal method. In the intraoral method a small piece of film (which is light and moisture-proof) is placed over the gum tissue at a point where trouble is suspected, placing the tube in such a position that perpendicular rays will be cast on the teeth and film. This method covers a smaller area, but produces a picture with very sharp details, and is especially recommended for anterior teeth. The extraoral or buccal method requires that a plate 5 x 7 be brought in contact with the jaw at the region of the suspected trouble. The patient inclines the neck and head to an angle of about forty-five degrees. The tube is placed over the shoulder to the opposite side at a distance of twenty inches from the face to avoid superimposition of the jaws. This process produces a picture of greater area and is intended for bicuspids and molars. Two skiagraphs are taken from two angles or directions to determine whether we have a buccal, lingual, distal, or mesial presentment, and I might mention here that Dr. Cryer suggested that I make stereoscopic skiagrams which permit viewing by a reflecting stereoscope, and as a result, instead of observing flat pictures, we obtain a relief or stereoscopic perspective effect, which shows the exact position of the teeth. I have received very good results with my stereoscopic skiagrams at the Philadelphia Hospital, where I used a special table which I had built for the purpose. There is no danger of X-ray burns in skiagraphing these conditions; the time of exposure is short, being from one second to two minutes.

*Dr. T. C. Stellwagen,* Philadelphia.—The important illustrations made by Dr. Cryer explain how great damage has been done by meddlesome dentists. "Orthodontia" is an incorrect term; the correct one would be "taxidontia." By attempts to correct dental irregularities without a proper knowledge of the development of the jaws an amount of mischief has been done which the profession does not take into account. Early in my practice there occurred a number of instances of patients saying that they had had irregularities corrected, and that since then they had suffered more or less pain and soreness about the teeth; conditions resembling those following fracture or sprain when the weather seems to bring about a neuralgic state. Very often the violence used for such correction has been sufficient to cause permanent inconvenience. Many of the cases, credited to the efficiency of the appliances used, would have been better if left to Nature. Dr. Cryer shows us that the development and growth of the jaws are not confined to any particular locality, but are general throughout, and that under peculiar circumstances we find certain centres of growth arrested, just as we see in hip-joint disease, and then permanent deformity results. The interesting question in connection with the case of the Baltimore child is, can we awaken that trophic influence? Some years ago the permanent cuspid teeth of a patient had failed to erupt, still the deciduous cuspid teeth were *in situ* to work on. Irritation of the teeth until they evinced considerable pain and soreness failed. Ligatures tied around them, hoping thereby to revive or reawaken this trophic force, failed. Extraction of one deciduous canine failed. So far there is but little, if any, hope of ever stimulating these trophic centres. The case mentioned is of interest, as the lad had spasms, caused seemingly by the canine pressure on filaments of nerve in the jaw. The spasms came only occasionally, it is true, but so growth has remissions of energy. I believed that we could do nothing better than to look forward to the time when the growth and alterations of the permanent canine teeth would cease, somewhere about his twelfth year. For about forty years, although still living, the patient has been free from spasms. That the reawakening of the trophic force could have been brought about in his case I very much doubt.

*Dr. Bogue.*—In a case of a child twelve years of age, in whom the left central incisor had not developed, instead of tapping on that bicuspid tooth, as did Dr. Stellwagen, I put in wedges and spread the right central and left lateral widely apart, and inserted a large central on a rubber plate. The left central came down, even at that age, into correct position. I have done the same thing in the last six months. In other words, I got the room which Dr. Cryer has been telling us we ought to get. The plaster cast of impaction shown us by Dr. Cryer shows also a retracted condition of the incisors, and I think he is mistaken as to the cause. It seems presumptuous for me to question Dr. Cryer, but he must not draw conclusions from the facts he presents to us, unless he expects to be criticised. The irregularities of the incisors in that case seem to me to have nothing to do with the other irregularities which he showed us.

*Dr. Stellwagen.*—There was abundant room for the cuspids to come down, the teeth were separated and had enough space.

*Dr. Eugene S. Talbot,* Chicago.—Dr. Cryer's specimens are fine and show many pathologic conditions. The explanation of an individual case does not, however, give accurate information as to the etiology. To understand the etiology, the evolution of man, including heredity and neurasthenia in the parent as well as in the child, must be considered to show the production of these conditions. If there were time, I should like to go over these slides and from these basic principles show how these deformities are produced. Starting with the evolution of man, the face and jaws, like the vermiform appendix, small rib, little toe, and other structures of the body are degenerating under the law of economy of growth for the benefit of the organism as a whole. At about four and one-half months of fetal life, the first period of stress occurs. At this period, owing to the neurasthenia of the parents and improper nourishment, arrests take place. Owing to the transitory nature of the face and jaws, they are more easily affected than permanent structures of the body. Arrests of development of some parts and excess of development of others (due to an unstable nervous system) account for all malformations. The point brought out by Dr. Stellwagen in regard to pain in the alveolar process is not uncommon. It occurs after teeth have been regulated, and the question naturally arises when and how far we are warranted in the correction of irregularities, owing to the interstitial gingivitis produced. This pain occurs in ordinary forms of interstitial gingivitis, as well as in interstitial gingivitis due to injury. This disease is associated with the alveolar process through life. Pain may be experienced at any period. The cribbing of the horse when returned to the stall after a summer's outing and disuse of the anterior teeth is a marked illustration of this pain. The eruption of the cuspid tooth seems to be a providential process by which the anterior and posterior teeth are, in a measure, moved forward or backward for the purpose of enlarging the dental arch.

*Dr. Cryer.*—In regard to the use of the term "premolar," I perhaps should not use this word before a dental society, as I believe they have arbitrarily adopted another term, which, however, I can not accept in my writing. A premolar is a tooth anterior—in time or position—to a molar tooth. Dr. Bogue says we have not a deciduous premolar. In one sense, all the deciduous teeth are premolars, but the term is more especially and more properly applied to what are commonly called, by the dental profession, the deciduous molars. If I understand the meaning of deciduous, it means not lasting but destined to be lost or shed in time. The deciduous (premolars) molars are shed and their places taken by the permanent premolars, or as they are known in accepted dental nomenclature, the bicuspids. The lower jaw certainly grows forward. The upper jaw I claim does not grow forward, but its alveolar process does. It extends forward, and its base is the upper jaw. The upper jaw is one thing; the alveolar process and its teeth are distinctly another. When teeth are lost, and the alveolar processes are resorbed, the rim of the lower jaw, which has gone forward, remains there, while the upper jaw, not having gone forward, is found to be almost the same as in early life. I want to put it on record that four years ago the city authorities of the Philadelphia Hospital recognized the dental profession by adding four doctors of dental surgery to the medical staff of that great hospital, giving them the same standing as the medical men.

Last year, at the suggestion of the dental staff, an extensive apparatus for X-ray work was added to the already well-supplied hospital, and the dental department was put in the highest possible condition for usefulness. Dr. Kassabian has spared neither time nor energy in working up the use of the X-ray for the benefit of the dental students who have attended the dental clinics given at the hospital.

## Figures and Tables

**Fig. 1. f1:**
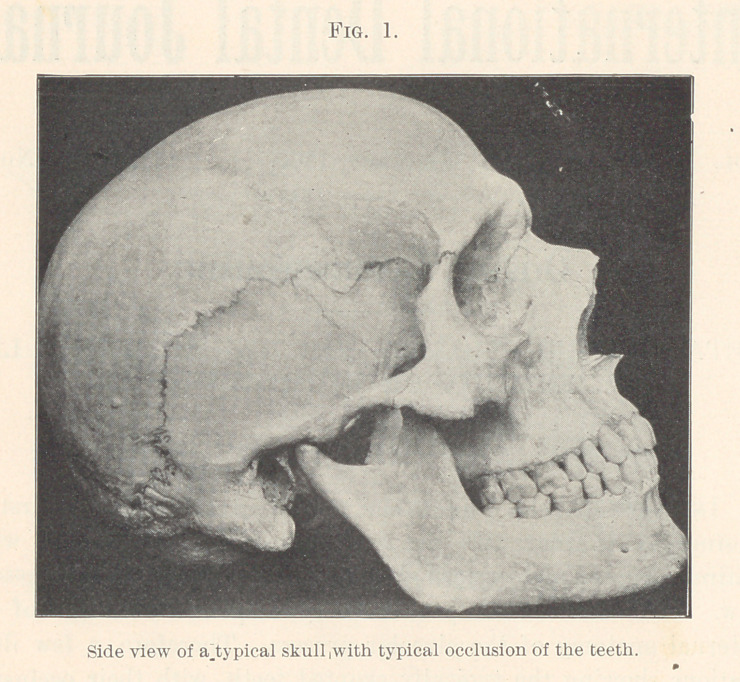


**Fig. 2. f2:**
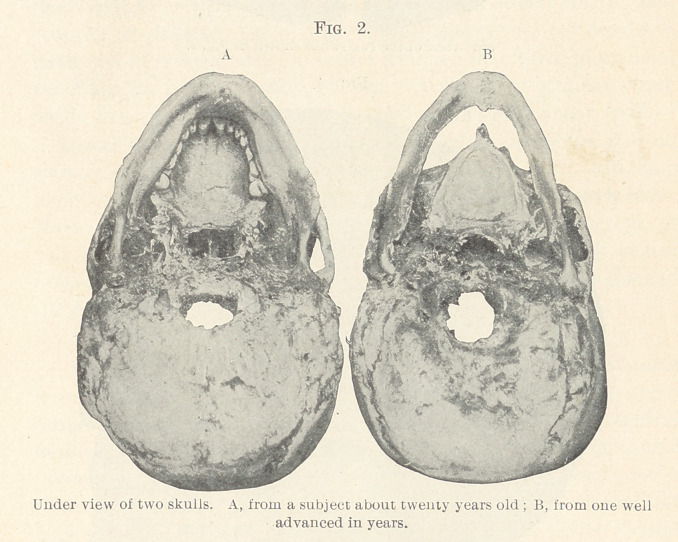


**Fig. 3. f3:**
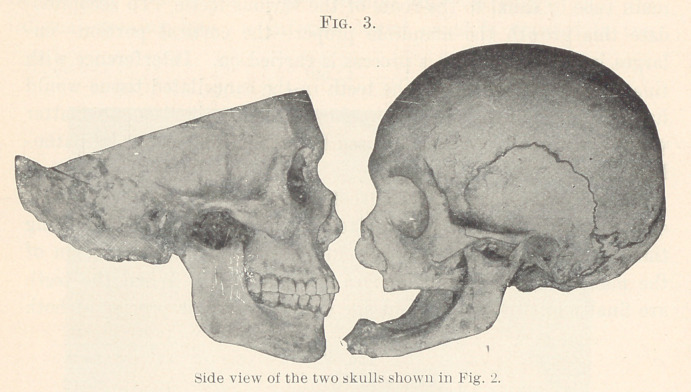


**Fig. 4. f4:**
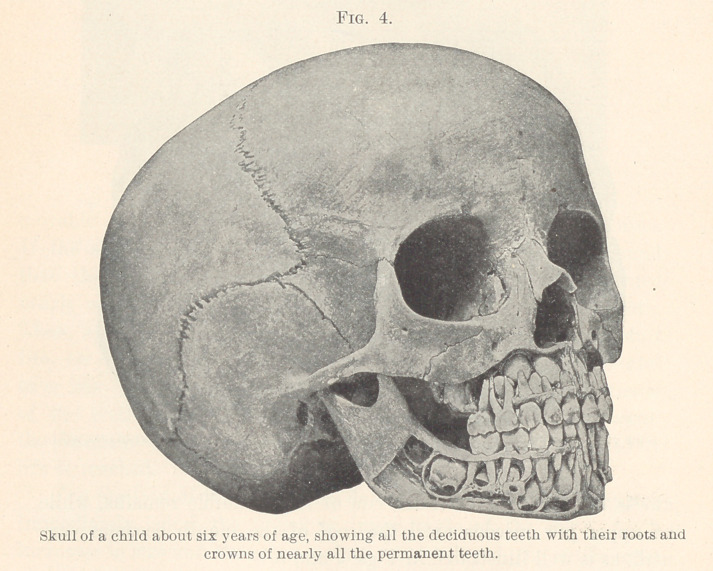


**Fig. 5. f5:**
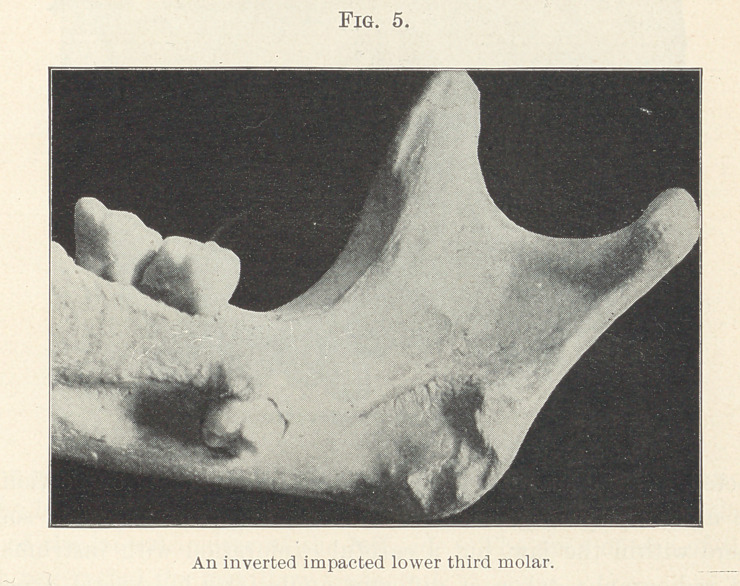


**Fig. 6. f6:**
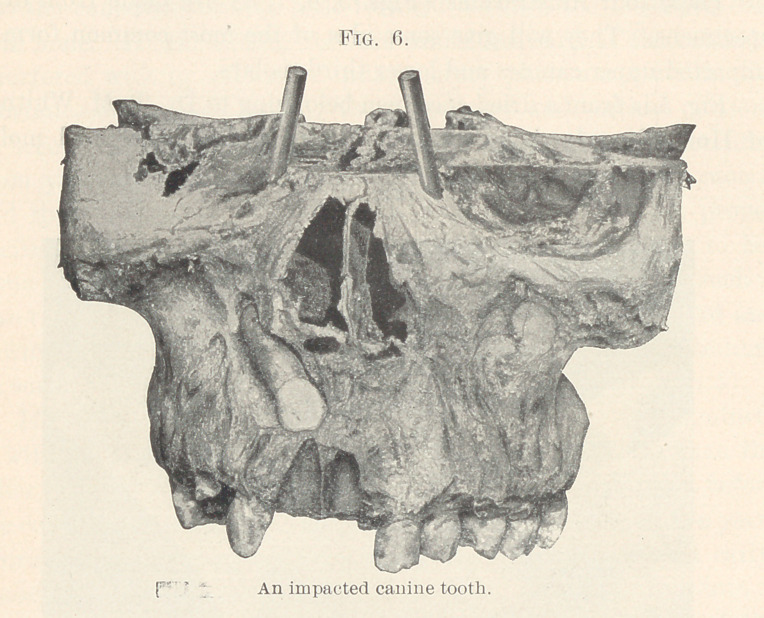


**Fig. 7. f7:**
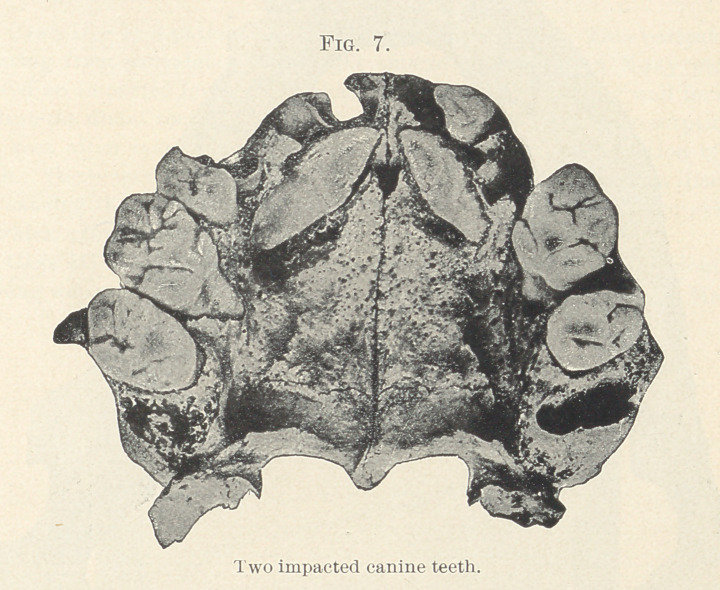


**Fig. 8. f8:**
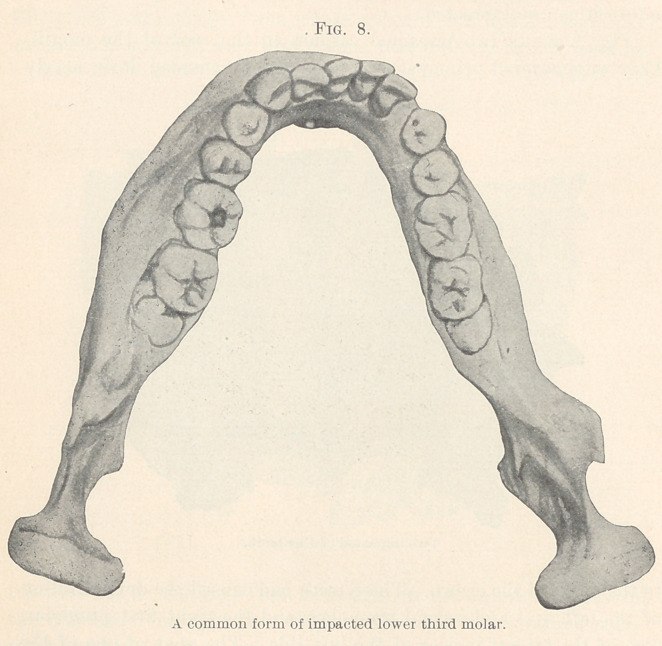


**Fig. 9. f9:**
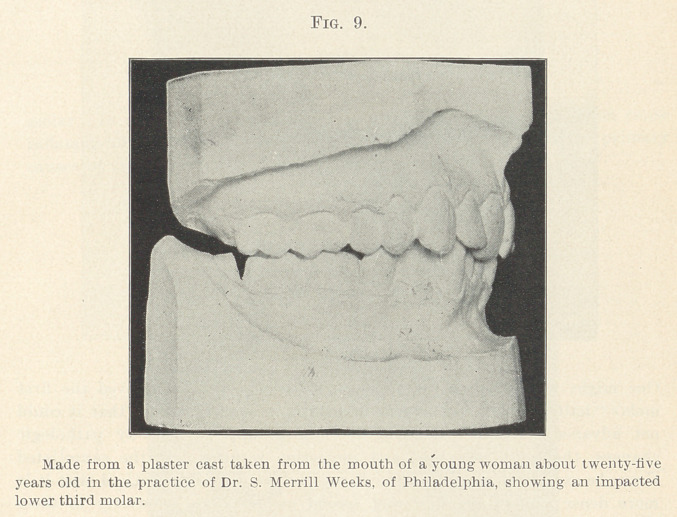


**Fig. 10. f10:**
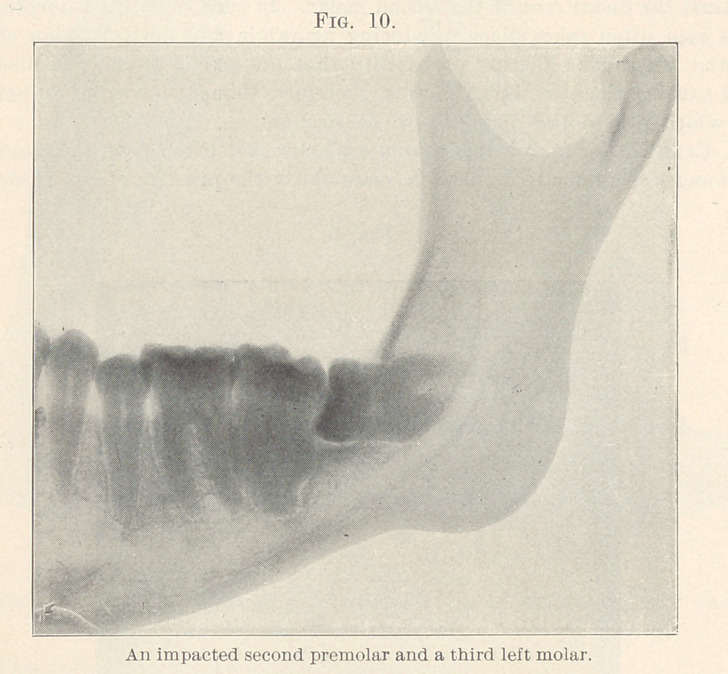


**Fig. 11. f11:**
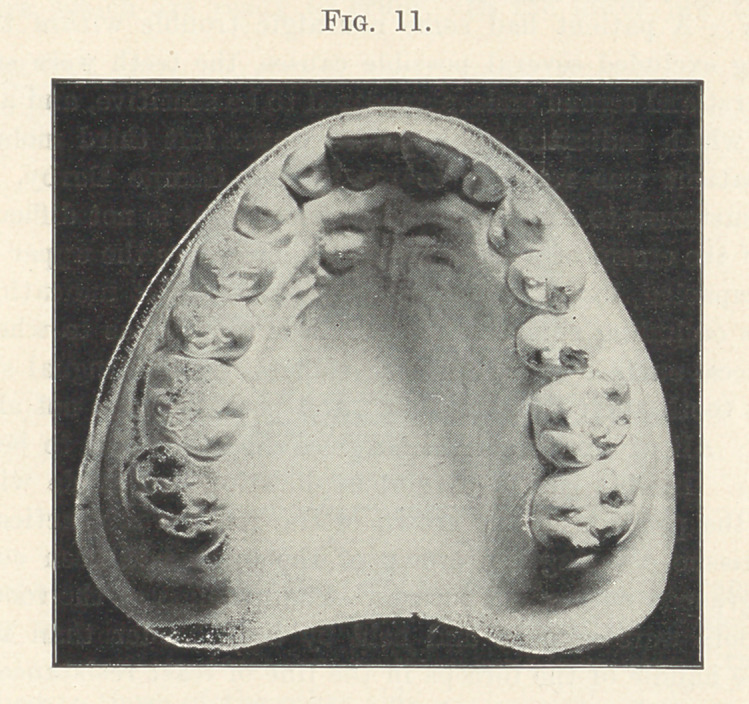


**Fig. 12. f12:**
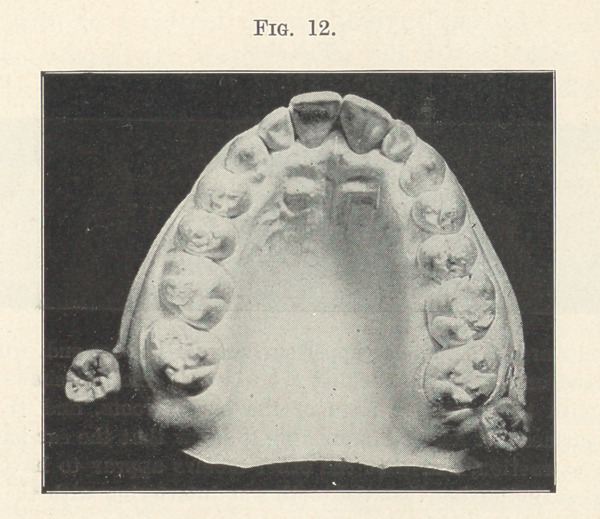


**Fig. 13. f13:**
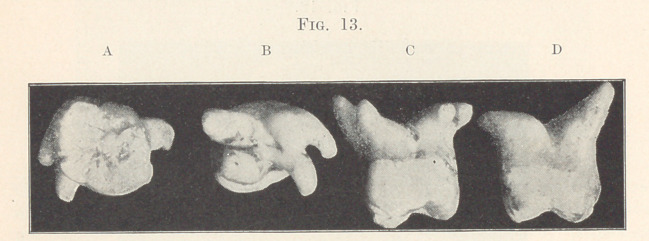


**Fig. 14. f14:**
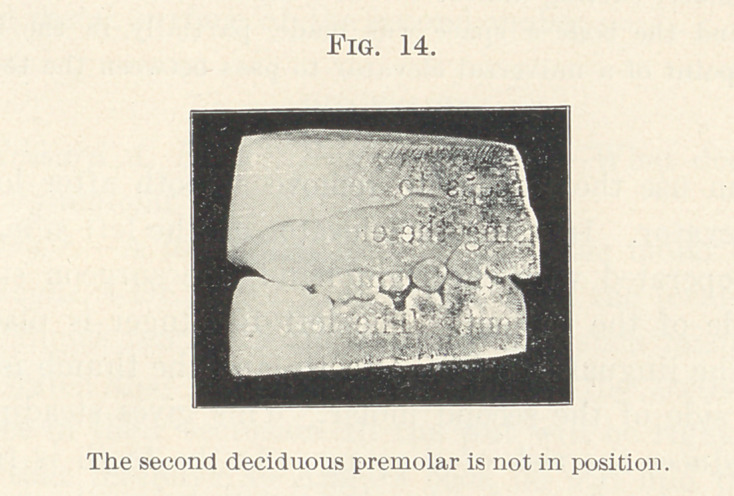


**Fig. 15. f15:**